# Two-photon polymerisation 3D printed freeform micro-optics for optical coherence tomography fibre probes

**DOI:** 10.1038/s41598-018-32407-0

**Published:** 2018-10-04

**Authors:** Jiawen Li, Peter Fejes, Dirk Lorenser, Bryden C. Quirk, Peter B. Noble, Rodney W. Kirk, Antony Orth, Fiona M. Wood, Brant C. Gibson, David D. Sampson, Robert A. McLaughlin

**Affiliations:** 1 Australian Research Council Centre of Excellence for Nanoscale BioPhotonics, Adelaide Medical School, The University of Adelaide, Adelaide, SA 5005 Australia; 2 Institute for Photonics and Advanced Sensing, The University of Adelaide, Adelaide, SA 5005 Australia; 3 Optical + Biomedical Engineering Lab, University of Western Australia, Perth, WA 6009 Australia; 4 Cylite, Notting Hill, VIC 3168 Australia; 5 Centre for Neonatal Research and Education, The University of Western Australia, Perth, WA 6009 Australia; 6 School of Human Sciences, The University of Western Australia, Perth, WA 6009 Australia; 7 Australian Research Council Centre of Excellence for Nanoscale BioPhotonics, RMIT University, Melbourne, VIC 3000 Australia; 8 School of Surgery, The University of Western Australia, Perth, WA 6009 Australia; 9 Department of Electrical, Electronic and Computer Engineering, University of Western Australia, Perth, WA 6009 Australia; 10 The University of Surrey, Guildford, Surrey, GU2 7XH United Kingdom

## Abstract

Miniaturised optical coherence tomography (OCT) fibre-optic probes have enabled high-resolution cross-sectional imaging deep within the body. However, existing OCT fibre-optic probe fabrication methods cannot generate miniaturised freeform optics, which limits our ability to fabricate probes with both complex optical function and dimensions comparable to the optical fibre diameter. Recently, major advances in two-photon direct laser writing have enabled 3D printing of arbitrary three-dimensional micro/nanostructures with a surface roughness acceptable for optical applications. Here, we demonstrate the feasibility of 3D printing of OCT probes. We evaluate the capability of this method based on a series of characterisation experiments. We report fabrication of a micro-optic containing an off-axis paraboloidal total internal reflecting surface, its integration as part of a common-path OCT probe, and demonstrate proof-of-principle imaging of biological samples.

## Introduction

Optical coherence tomography (OCT) is an optical medical imaging technique that provides cross-sectional structural images with a high axial resolution (better than 20 µm) and high sensitivity to reflectivity (over 100 dB)^1,2^. However, OCT suffers from shallow depth penetration in highly scattering biological tissue (typically 1–3 mm). This has limited the use of conventional bench-top OCT systems to largely transparent tissues, such as the eye, or superficial tissue layers, such as the skin. Imaging other tissues has required the development of miniaturised probes, typically for endoscopic use^2,3^, intravascular use^4,5^ or, in solid tissue, by encasing the probe in a needle^6,7^. These miniaturised fibre-optic imaging probes enable us to see deep inside the body *in vivo* with micron-scale resolution, and have deepened our understanding of many diseases and disorders^3–13^.

There are two common methods to fabricate miniaturised fibre-optic probes. The first is to assemble the probe from discrete micro-optical elements (e.g., GRIN or ball lenses, and prisms or mirrors). However, manufacturing such glass micro-optics requires special handling tools (for blocking, grinding and polishing, etc.) and is a time-consuming process^14^. Moreover, building a high-quality probe with glass micro-optics requires extremely precise alignment to minimise backreflection^15^. Alternatively, OCT probes can be created by fusion splicing fibre lenses, such as GRIN multimode fibre or fibre-based ball lenses, directly to the optical fibre. This method, demonstrated by our group and several others^6,7,16–18^, uses a splicer with automatic precise fibre alignment^18^. Such methods have not yet been shown capable of producing a micro-optic with a complex geometry, beyond simple few-element symmetric designs.

Here, we propose and demonstrate the use of a novel technology to fabricate micro-optic OCT probes—3D laser lithography. This 3D printing technology is based on two-photon direct laser writing (TPDLW) and has demonstrated the potential to print complex freeform optics with sub-millimetre dimensions^19–24^. By utilizing two-photon absorption to perform highly localised polymerisation in the focal volume, this technology enables fabrication of near-arbitrary 3D micro/nanostructures with a spatial resolution in the range 80–500 nm and sub-wavelength (~15 nm) roughness on the optical surface^19,20^. In this paper, we report our progress in utilizing a commercially available TPDLW system (Photonic Professional GT, Nanoscribe GmbH, Germany) to rapid-prototype miniaturised optics for OCT fibre probes. We comprehensively evaluate the capability of this fabrication method, in reporting a series of characterization experiments. To demonstrate the potential applications, a fully functional OCT probe with a 3D printed optic was fabricated and successfully used for OCT scanning. The optic contains an off-axis paraboloidal total-internal-reflection (OAP-TIR) surface that can both focus and redirect the beam. This probe also includes a common-path interferometer configuration^1^, conveniently avoiding the need to carefully match the optical pathlengths of separate sample and reference arms.

To the best of our knowledge, this is the first application of TPDLW 3D printing technology in OCT, and demonstrates the feasibility of rapid-prototyping highly compact freeform optics for OCT fibre-optic probes.

## Results

To characterise the optical properties of 3D printed micro-optics, we performed experiments to measure the optical attenuation, and characterise the ability to redirect the light beam over the OCT operating wavelength range, 1235–1365 nm.

As all 3D printed samples were fabricated using a layer-by-layer TPDLW process, a large layer thickness induces high surface roughness^21^. When the surface roughness is large (i.e., over one tenth of the wavelength), these irregularities on the surface will behave as a grating and diffract the beam^25^. Thus, we utilised a small layer thickness (100 nm) to ensure low surface roughness similar to previous work^19^. To evaluate the manufactured quality of 3D printed samples, we imaged all printed optics using an environmental scanning electron microscope (SEM). In addition, we profiled the beams exiting the 3D printed micro-optics over the OCT operating wavelength range to evaluate the overall quality of each printed optic.

To demonstrate that 3D printed optics can be made into a working OCT probe, we designed and fabricated an optical assembly for side-viewing OCT imaging. The probe was coupled to an OCT system, and was used to image microstructure in biological samples.

### Characterization: Optical attenuation of a 3D printed optical element

**Figure 1:**
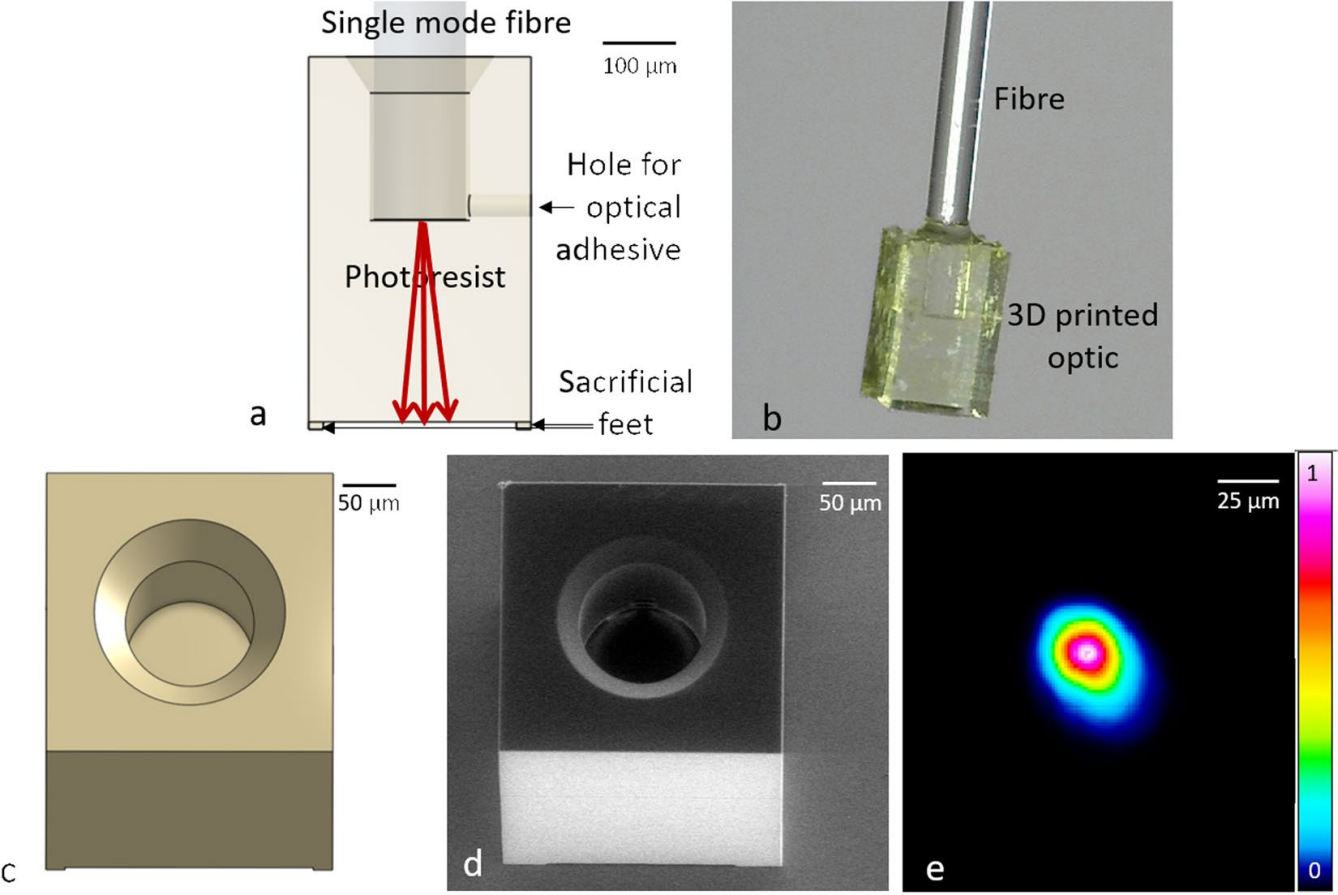
(**a**) Transparent front view of the flat-end block and fibre assembly. Red rays indicate propagation of the exit beam. (**b**) Microscope image of the printed structure assembled with a fibre. (**c**) 3D perspective view of the design. (**d**) SEM image of the 3D printed structure. (**e**) Profile of the beam after transmission through the block with a colour map of intensity (A.U.) in linear scale. The beam was imaged at approximately 50 µm from the surface of the flat-end block.

OCT probes must have very low optical losses in order to provide sufficient sensitivity to effectively image biological samples. We characterised the optical loss incurred by a 3D printed optical element using a simple flat-end block – see Fig. 1(a). A fibre slot was designed in this block, to allow the structure to be mounted on the fibre for characterization, as shown in Fig. 1(b). Optical adhesive was then applied to affix a fibre in the slot. Note that an additional hole connecting the fibre slot to the outer surface of the printed structure was also included in the design, so as to allow the adhesive to readily flow through the entire fibre slot. Four ‘sacrificial feet’ were also created, with which the sample could be easily detached from the glass substrate at the completion of printing, without damage to the main structure.

The SEM image of the flat-end block (Fig. 1(d)) demonstrates that such a printing technique can produce a structure with high manufacturing quality. To measure the beam profile, light from a fibre-coupled OCT light source was coupled through a single-mode fibre patch cable and into the flat-end block. We compared the power of the incident light upon the 3D printed optical block (i.e., the light directly exiting the fibre patch cable) to the light transmitted through the 3D printed optical block (i.e., the light exiting the block which has been mounted onto the single-mode fibre), as measured with a complementary metal-oxide-semiconductor (CMOS) camera. This measurement provided us with an estimate of 4.6% for the average optical attenuation of the 3D printed optical element (chief ray travels 270 µm in the element) over the wavelength range used for OCT imaging. By comparing the losses for two flat-end blocks of different lengths (chief-ray path lengths 270 µm and 180 µm), we estimated the loss per unit length to be 0.68 dB/mm. Such low loss indicates that photoresist could be a suitable material from which to fabricate micro-optics for OCT applications. Note that this measured loss is a combination of absorption by the photoresist material, scattering in the bulk and at the interfaces, and loss caused by the Fresnel reflection between fibre/glue/photoresist. The profile of the beam captured by the camera after passing through the block is shown in Fig. 1(e), showing an undistorted Gaussian profile similar to that for the beam exiting the fibre, and without evidence of any diffraction pattern. Such artefacts could arise if there were sufficient surface roughness or if there were inhomogeneities in the refractive index caused by variations in the degree of cross-linking of the photoresist^26^.

### Side-viewing probe: Redirecting a beam by total internal reflection

To design a side-facing OCT probe based on total internal reflection, the value of the refractive index of the 3D printed optical material must be estimated. Gissibl *et al*. have measured the refractive index of the UV polymerised photoresist in the visible spectral range^27^. Based on these results, the refractive index of the cured photoresist at 1300 nm is calculated to be 1.494 using Cauchy’s equation. For this refractive index, a critical angle of 42**°** is needed for total internal reflection (TIR).

**Figure 2:**
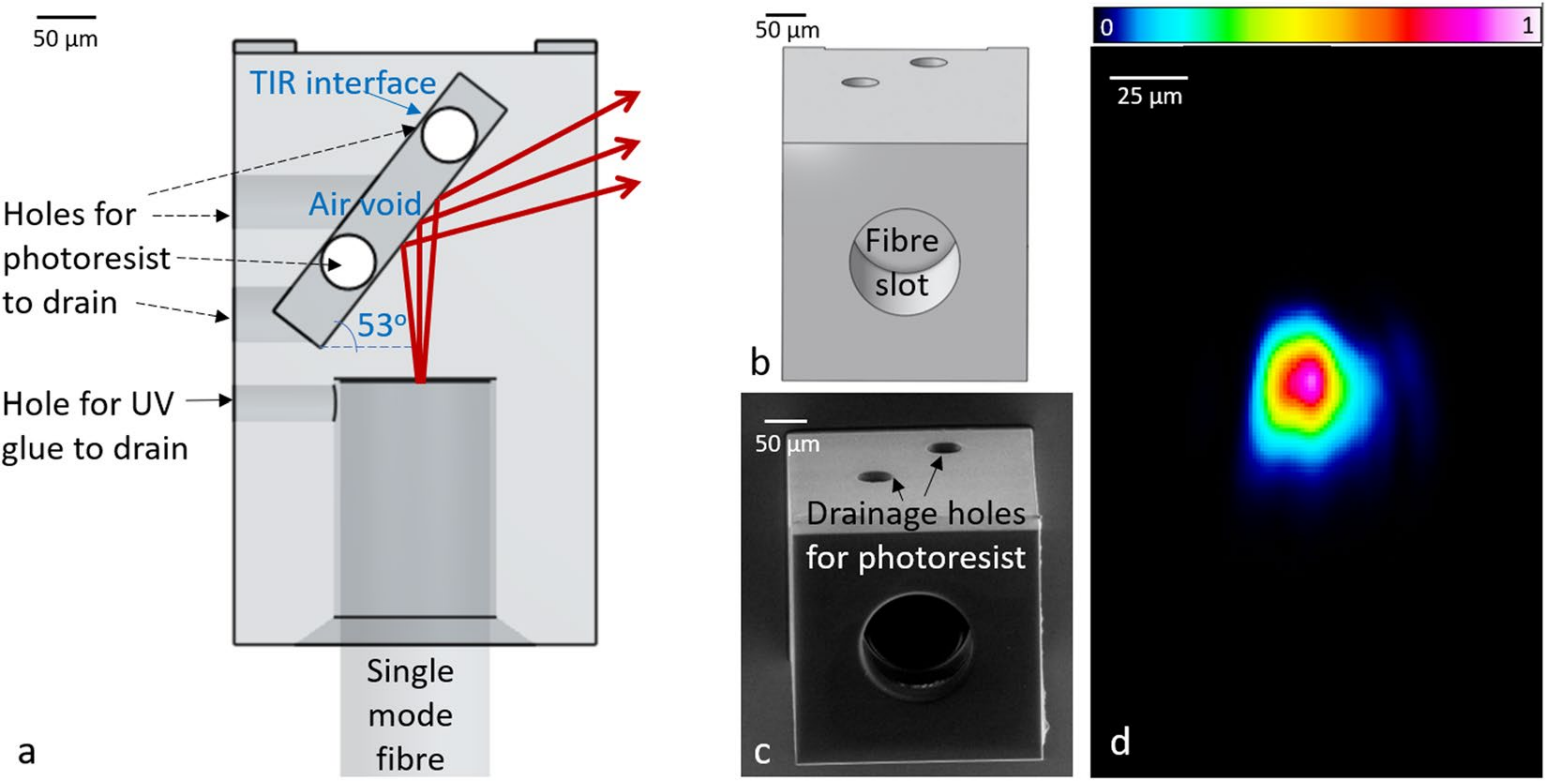
(**a**) Transparent front view of the TIR optic and fibre assembly. Red rays indicate beam propagation. (**b**) 3D perspective view of the design. (**c**) SEM image of the 3D printed TIR optic. (**d**) Profile of the beam after transmission through the TIR optic from a plane approximately 50 µm from where the light exits the 3D printed structure, with a colour map of intensity (A.U.) in linear scale.

We designed a structure with a tilted air void (see Fig. 2(a)) to generate TIR at the photoresist-air interface. A tilt angle of 53° was chosen so as to be sufficiently greater than the critical angle to safely account for any changes in the refractive index of the photoresist due to variations in the degree of polymer cross-linking^26^, and to ensure TIR for all rays in the divergent cone of light emitted by the fibre. The design and SEM image of the 3D printed TIR optic are shown in Fig. 2(b,c), respectively. As an air-filled void is required internally within the printed structure to maintain the interface between photoresist and air for TIR, we included four holes connecting the air void to the outer surface of the structure to create outlets for uncured photoresist to exit during 3D printing. The profile of the beam after being redirected by the TIR optic is shown in Fig. 2(d). Some minor beam distortions are visible, but no obvious diffraction patterns can be seen. Ninety four percent of incident light was measured at the reflection side of the structure by the beam profiler, whilst no light was measured at the other sides of the structure. Of the 6% loss, 4.6% could be attributed to attenuation, as measured in the structure in Fig. 1, in which light travels the same distance in the photoresist as in this TIR optic. This observation suggests that the design effectively achieves TIR.

### Fabricating a side-viewing imaging probe: Complete probe design

**Figure 3:**
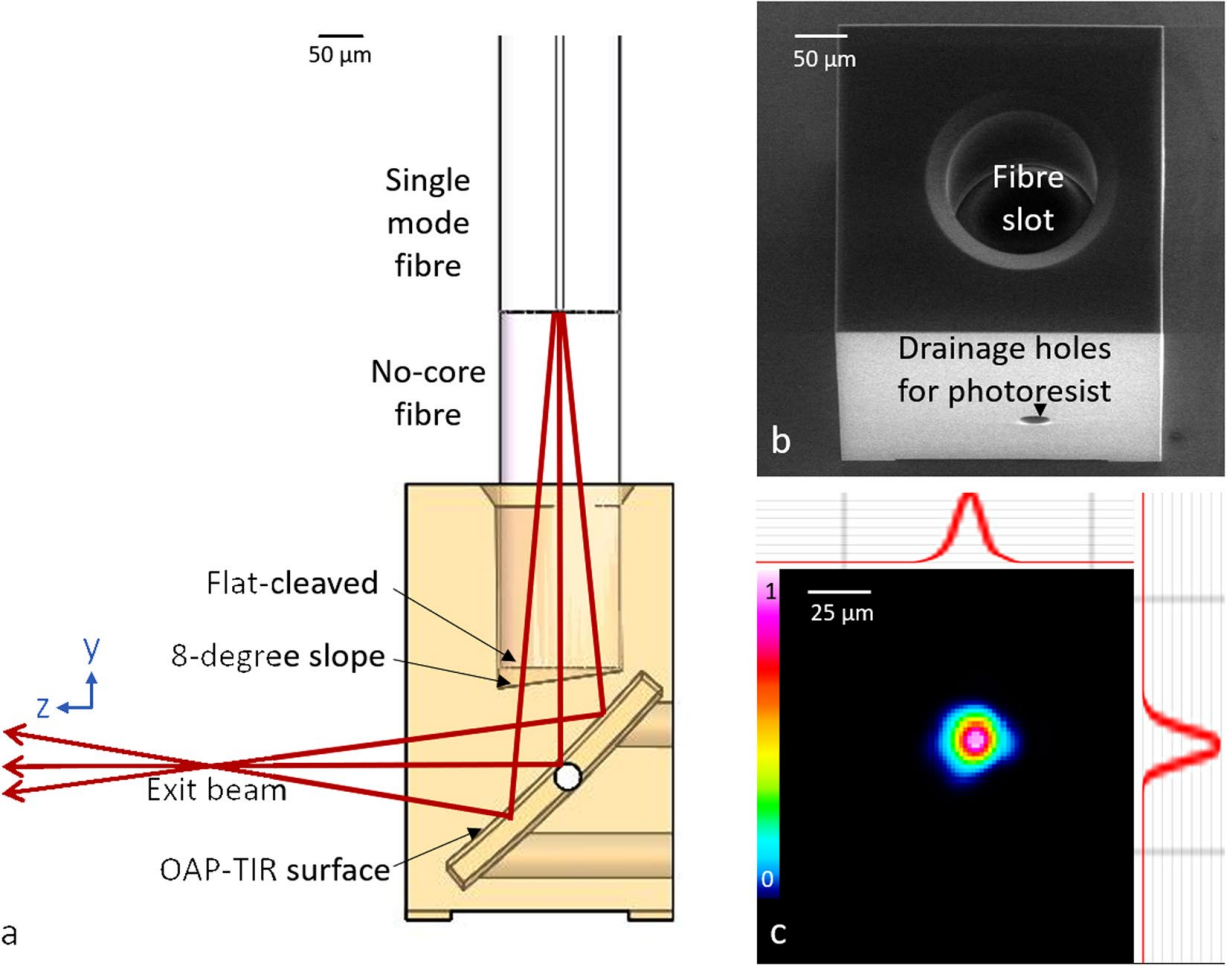
(**a**) Transparent front view of the OAP-TIR optic and fibre assembly. Red rays indicate beam propagation. The reflection generated at the interface between the flat-cleaved fibre and air is used to create a reference signal for OCT imaging. Note that the z-position of the focal point shown in the schematic is for illustrative purposes and is not drawn to scale. (**b**) SEM image of the 3D printed OAP-TIR optic. (**c**) Profile of the beam at the focus of the OAP-TIR optic at approximately 800 µm after exiting the 3D printed optic. The top and right insets are cross-sections of the beam profile through the centre. The colour map and plots of intensity (A.U.) use a linear scale.

To demonstrate the potential application of this technology, a fully-functional OCT probe utilising a 3D printed optic was fabricated and successfully used for OCT scanning. In this probe, focusing and redirecting the OCT beam were achieved by using an OAP-TIR surface schematically shown in Fig. 3(a), designed using optical design software (OpticStudio, Zemax LLC., USA). A single-mode fibre was spliced to a 600-µm section of no-core fibre, in order to expand the beam to fill most of the aperture of the OAP-TIR surface, prior to focusing, to enable a longer working distance. As indicated by the beam profile, Fig. 3(c), the lateral resolution (i.e., 1/e^2^ beam diameter) is approximately 23 µm.

**Figure 4:**
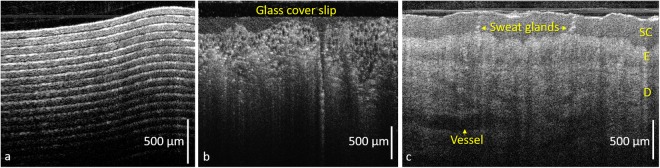
OCT cross-sectional images of: (**a**) tape phantom; (**b**) cucumber; and (**c**) *in vivo* scan of a human palm using the common-path 3D printed OAP TIR fibre assembly. SC: Stratum Corneum; E: Epidermis; D: Dermis. The axial scale bars show optical distance.

The spliced fibre tip was inserted into the fibre slot in the OAP-TIR optic. Importantly, this tip was flat cleaved to create a reference reflection for OCT imaging, enabling a common-path OCT configuration. The measured reflection generated at this fibre-to-air interface was 0.15%, which is comparable to that of the reference arm of our commercial OCT system (Telesto III, Thorlabs GmbH, Germany) when operating in dual-arm mode with the same A-scan exposure time. The flat-cleaved design, with a 600-µm section of no-core fibre, was chosen to ensure sufficient reflection for good OCT sensitivity^28^. To reduce subsequent parasitic reflections, the distal end of the fibre slot was printed with an 8-degree slope (Fig. 3(a)). The common-path configuration avoids the need to modify the optical pathlength of a separate reference arm when changing between probes with different optical pathlengths. This probe was mounted on a motorised linear translation stage to scan and produce 2D B-scan images. The OCT images (Fig. 4) show the internal structure of a tape phantom (multiple layers of adhesive tape on a glass slide), a section of cucumber and an *in vivo* scan of skin on a human palm, demonstrating the effectiveness of the overall system and the probe’s capabilities to resolve fine structures in the axial and lateral directions.

## Discussion

Most commonly, OCT fibre-optic probes are fabricated with either discrete micro-optics^2^ or made by splicing sections of different fibres^1^. The fabrication technology demonstrated in this report opens up new possibilities to rapidly prototype OCT probes that contain micro-optics with complex geometries and functions, especially microscale freeform optics that were previously infeasible. The OCT micro-optic designed in this study utilised an off-axis paraboloidal total internal reflection surface to focus the beam and redirect it to achieve side viewing, and an 8-degree-angled air-to-photoresist interface to reduce parasitic backreflection and, thus, undesired additional common-path reference signals. Other designs, such as structures that achieve freeform beam shaping^20^, as well as designs that simultaneously extend the depth of focus^17^, and avoid astigmatism^29^ are possible. Special micro-optic elements may also be fabricated with these technologies, such as GRIN lenses^26^. In addition, new materials are extending the use of 3D printed optics to areas such as high-intensity optics^30^.

As this is the first demonstration of TPDLW 3D printing technology in an OCT application, we have focused on evaluating the appropriateness of the technology for fabricating OCT micro-optics. In this study, we characterised key properties, such as the optical attenuation of the cross-linked photoresist in a commonly utilised OCT wavelength band (around 1300 nm).

The commercially available 3D printer used here is limited to fabricating structures as a combination of contiguous 300 µm blocks using a high-precision piezoelectric translation stage, and inhomogeneities may be present at the interfaces between these blocks^31^. Limiting the OCT focusing optics to less than 300 µm thickness can restrict the beam properties of the probe^15^, such as lateral resolution and the inclusion of components to redirect the light beam. Thus, to demonstrate the suitability of this technology for OCT imaging, we have fabricated optics on a scale with dimensions larger than 300 µm. Previous studies have fabricated small lenses (less than 300 µm) using this printer^19,32^. We enabled the fabrication of larger micro-optics by utilizing a “dip-in” lithography technique and by stitching together multiple contiguous subcomponents, where the length containing the optical path is no more than 300 µm to maintain high optical performance.

**Figure 5:**
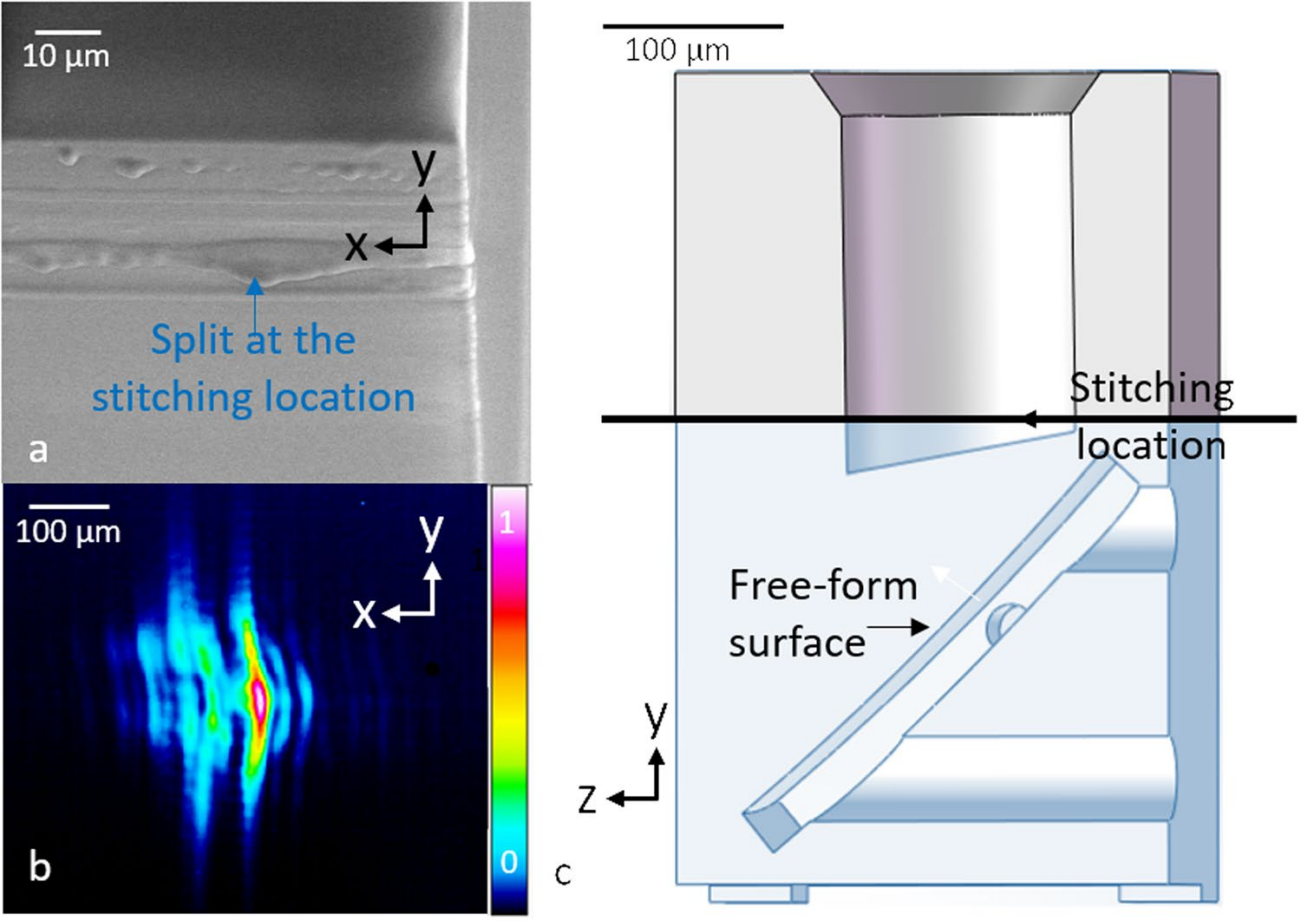
The effect of stitching. (**a**) SEM image of the split generated at a stitching region. (**b**) Beam profile when a stitch is present in the optical path. (**c**) The boundary of grey and blue regions indicates the stitching location of the OAP-TIR design, where we strategically arrange the split in a region that is not in the optical path. Note that the surface distortion in (**a**) is magnified due to the charging artefact generated at the stitching location during SEM imaging.

The dip-in lithography technique is implemented by directly dipping the objective lens into a photoresist, which serves as both the immersion and photosensitive medium simultaneously, so as to increase the height of an object that can be printed. In addition, we employed a vertical stitching technique^31^ to create OCT micro-optics larger than 300 µm in height by stitching together multiple contiguous subcomponents by the movement of the microscope’s z-translation stage. The drawback of this technique is that it generates an inhomogeneity at the interface of the 300-µm blocks (Fig. 5(a)) resulting in the distortion of the beam. As shown by Fig. 5(b), diffraction can be clearly observed in the beam profile when there is a split in the optical path. In the complex optic that was demonstrated in this work, we purposely designed the component so that the split occurred in a region that did not include the optical path, as shown in Fig. 5(c). Therefore, good image quality was maintained.

As the range of piezo stage movement within the 3D printer can potentially be increased^33,34^, an OCT micro-optic with diameter or height greater than 300 µm may, in principle, be 3D-printed without stitching. In parallel, detailed investigations to improve the dimensional and shape accuracy of the two-photon polymerization technology have been explored^24^. With such foreseeable improvements, we believe that the value of this technology for fabrication of OCT micro-optics will continue to improve.

## Conclusion

In summary, we have demonstrated the first successful application of TPDLW technology to 3D printed freeform micro-optics for OCT fibre probes. The 3D printed micro-optic exhibited low optical loss in the common 1300-nm OCT working wavelength range. Total internal reflection was achieved using 3D printed micro-optics with specially designed freeform air-photoresist interfaces, enabling the creation of a side-viewing OCT probe. In particular, we designed and fabricated a micro-optic that contains an off-axis paraboloidal total internal reflection surface, incorporated this into a common-path side-viewing OCT probe, and demonstrated OCT imaging of biological samples.

## Methods

### Micro-optics fabrication

The micro-optics were fabricated using a commercially available two-photon direct laser writing system (Photonic Professional GT, Nanoscribe GmbH, Germany)^19,20^. OCT probes typically utilise a weakly focused beam (with a working distance over 0.5 mm and a beam waist smaller than 30 µm) to ensure reasonable lateral resolutions over a millimetre depth range^7^; thus, a larger physical aperture is needed for OCT optics than has been previously reported for 3D printed lens^19,20^. To print an object with dimensions greater than 300 µm, and without producing severe distortion at the edges, an objective with a relatively low magnification power (25×) was used for all designs^20^, creating a larger field of view than with a high magnification printing lens (63×). To print a tall object, the dip-in technique was employed, as described in the discussion section. All micro-optics described in this report were created using the dip-in technique and with a 25x objective for consistency. The IP-S photoresist (Nanoscribe GmbH, Germany) was used, both because it is suitable for dip-in mode printing and because it can achieve a smooth optical surface. The cross-linked photoresist has a manufacturer-stated hardness of 150 MPa and a Young’s modulus of 4.5 GPa after two-photon polymerization. A special glass substrate coated with indium tin oxide was used to generate sufficient difference in refractive index between the substrate and the photoresist (*n*_*substrate*_ − *n*_*resist*_ > 0.03) without leading to unwanted back reflections during laser exposure. By detecting the interference fringes caused by this interface of the substrate and the photoresist, the 3D printer was set to begin printing at the substrate surface.

Considering the trade-off between the printing speed and motion accuracy, we selected galvanometer-mirror continuous-scanning mode, a mode with a medium printing speed and a medium motion accuracy, to guide the laser beam in the lateral direction. To ensure the highest motion accuracy between each layer, we used the high-precision piezo-stage to move the sample in the z direction from one layer to another, with a layer thickness of 100 nm. Due to the limited movement range of the stage, the structure was written in two sections (as illustrated in Fig. 5). Within each section, printing was achieved by moving the high-precision piezo-stage without moving the microscope z-drive. To ensure high image quality, the splitting locations were positioned so as to avoid stitching in the optical path, as described in the Discussion section. Before fabricating each micro-optic, dose testing was performed so as to optimise laser power, power scaling and scanning speed for each design^31^.

The OAP-TIR micro-optic was designed with a curve equation of *z* = −*y *+ *ay*^2^ +*ax*^2^ in the coordinate system of Fig. 3 (z is the optical axis of the reflected beam), where x, y and z are in units of mm, and *a* = 0.7 mm^−1^. Note that the lateral resolution of this probe design can be tuned using the quadratic terms of the curve equation. However, working distance and lateral resolution are coupled, and higher lateral resolution (smaller focal spot diameter) can only be obtained at the cost of a reduced working distance. The printer parameters Piezo Settling Time, Scan Speed, and Laser Power were set as 100 ms, 50000 μm/s and 42%, respectively. Under these settings, fabrication of the OAP-TIR micro-optic required 14.5 hours.

After each micro-optic was printed, they were immersed in a bath of the SU8 developer (1- Methoxy- 2- propyl acetate) for development: simpler structures (as shown in Fig. 1) for 9 min; for more complex structures having holes and air voids (as shown in Figs 2 and 3), for 14 min. After development, each sample was rinsed in isopropanol for 2 min and dried under a gentle nitrogen blow. No further baking, polishing or other treatment was required.

We used SEM (FEI Quanta 450 SEM, ThermoFisher Scientific, USA) to evaluate the manufacturing quality of the 3D printed samples. We imaged our samples in low vacuum mode at 10–15 kV in order to scan the samples without requiring a metal coating in order to avoid any possible changes to the micro-optic’s surface.

### Beam profiling

**Figure 6:**
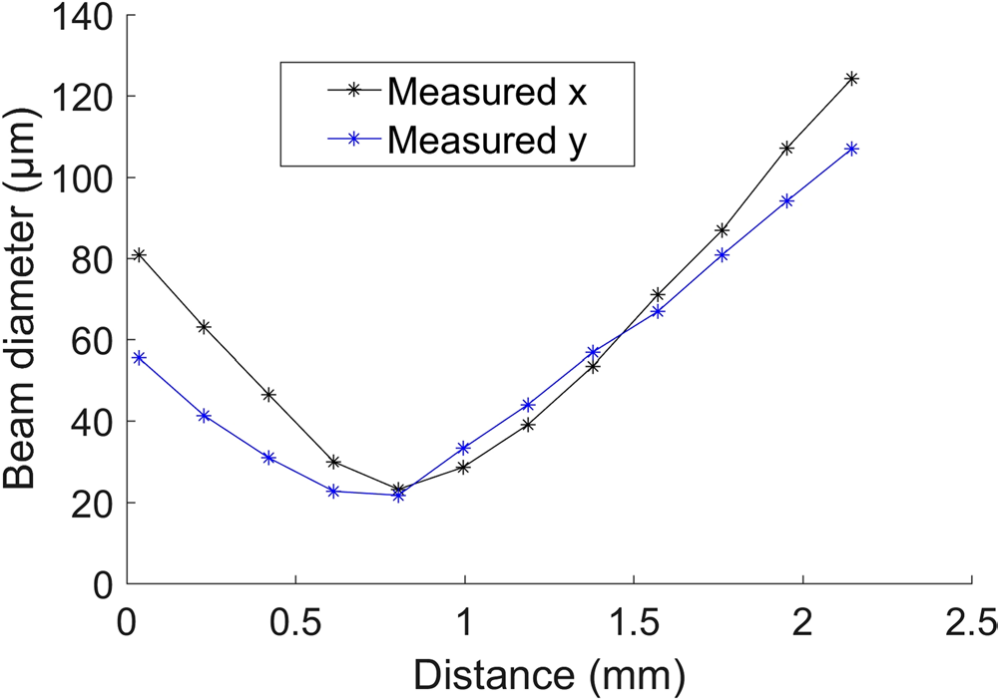
Beam profiles (1/e^2^) obtained from the off-axis paraboloidal TIR micro-optic whilst stepping through the beam waist with a linear translation stage (with a step size of 0.2 mm). The x and y axes are defined in the coordinate system shown in Fig. 3.

To measure the beam profile (Fig. 6), light from a commercially available spectral-domain OCT system (Telesto III, Thorlabs GmbH, Germany), with central wavelength of 1300 nm and bandwidth 136 nm, was coupled into the fibre that was interfaced to the 3D printed structure, see Fig. 1(b). The fibre assemblies were mounted onto custom designed holder-stage units to ensure that the optical beam from each fibre assembly was incident normal to the CMOS chip. Profiles of the beam were taken using a CMOS camera (WinCamD-XHR-1310, DataRay Inc., USA). The camera was moved axially by a linear translation stage (M2DU-50, DataRay Inc., USA) to step through the beam waist.

### Fibre probe fabrication

**Figure 7:**
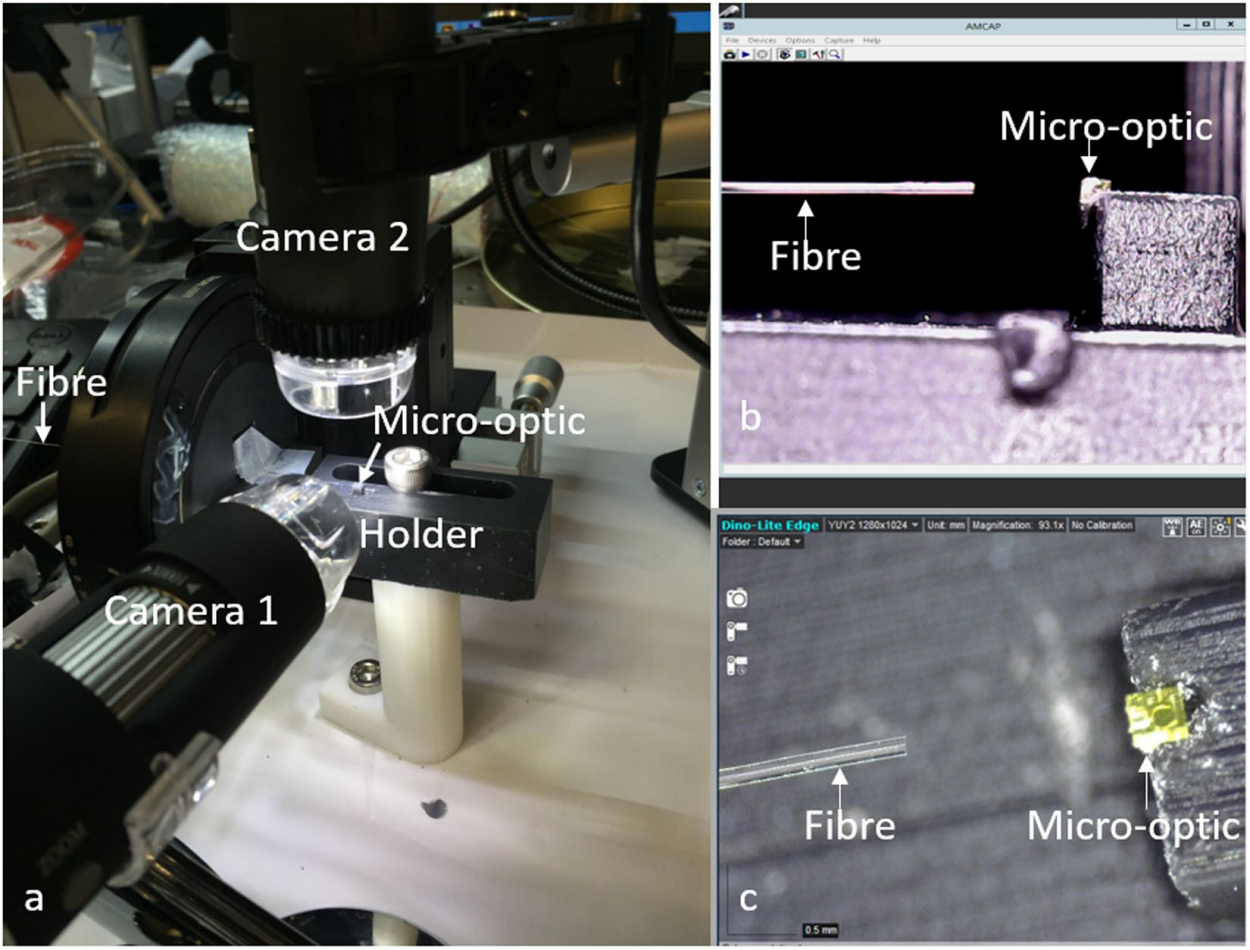
(**a**) Photo of the setup for aligning fibre and 3D printed micro-optic; (**b**) and (**c**) side-view and top-view video frames shown simultaneously by Camera 1 and 2, respectively, to guide the alignment.

We first spliced a 600 µm length of no-core fibre (NCF, POFC, Taiwan) onto an 80-cm length of single-mode fibre (SMF28, Thorlabs, USA). Subsequently, we added a drop of optical adhesive (NOA 83 H, Norland Products Inc., USA) around the no-core fibre close to (but not on) the distal end. The fibre was then inserted into the fibre slot of the 3D printed micro-optic. To ensure precise alignment of the fibre and the micro-optic, two holders, one for the fibre and one for the 3D printed optic (Fig. 7), were fabricated using a stereolithography 3D printer (Form 2, Formlabs Inc., USA) and controlled by translation stages during the alignment. The alignment process was guided by two video cameras to reveal the relative 3D positioning of the fibre and 3D printed optic, see Fig. 7(b,c). Once the fibre was inserted into the fibre slot, the adhesive between the distal end of the fibre and the micro-optic was cured by UV exposure.

### Imaging system setup

We used the light source, data acquisition card and workstation of a commercially-available OCT scanner (Telesto III, Thorlabs GmbH, Germany), with a manufacturer-specified axial resolution of 7.3 μm in air (with Hann Window to perform spectral shaping). This was interfaced to our OAP-TIR 3D printed OCT fibre probe (Fig. 3). A-scans were acquired at a rate of 28 kHz with an exposure time of 32 µs. B-scans were acquired using a linear pullback stage (M2DU-50, DataRay Inc., USA). A glass cover slip was positioned between the probe and the cucumber sample to achieve a flat surface.
